# Remarks on the Abortive Treatment of Typhoid Fever

**Published:** 1853

**Authors:** Stephen L. Burdett

**Affiliations:** Lancaster, Ky.


### WESTERN JOURNAL

OF

MEDICINE AND SURGERY.

---

NOVEMBER, 1853.

---

### ARTICLE I.—*Remarks on the Abortive Treatment of Typhoid Fever.*—By STEPHEN L. BURDEIT, M. D., of Lancaster, Ky.

I am aware how prone is the human mind to be captivated with novelties, and how new remedies, and new plans of combating disease, in numberless instances, have been extolled for a time, to be laid aside and forgotten. I am about to attempt to prove a thing which, by men high in our profession, has been pronounced impossible. I may be mistaken, but I have a sincere conviction that I have succeeded, in numerous cases, in cutting short typhoid fever. We ought not to be deterred from attempts to do this by the prejudices of the profession. Great names often do great mischief, by repressing enterprise and deterring from innovation. It is unfortunate when we settle down in the belief that no further improvements can be made in treating disease. There are some members of the profession who are ready to reject and ridicule any inroad upon the old and cherished ground, and also another set that are so bigoted and narrow minded, that they look upon the progression of the age with an eye of suspicion, and will not even give their attention to anything that makes pretensions towards advancement. Doctrines or theories that have stood the test of time and observation are, of late, being overturned and superseded by new ones, founded upon more extensive experience and observation—the only true test of any theory, doctrine, or principle. I do hope, and am very much inclined to believe, that the opinion so long taught and held, that Typhoid Fever will run a specific course, in spite of remedies, is one of them, and that it will some day only be remembered amongst the things that were, and as further evidence of the advancement of the age.

Wood, in his great work on Practice (3d edition, vol I., p. 339), asserts that it cannot be suddenly interrupted in its course, but admits that it can be very materially shortened and much influenced by treatment. These opinions of Wood do not argue any thing for the self-limited, or specific course of the disease, which was once, and is yet, so prevalent in the minds of our profession, and even of the community generally. I have heard it from the rostrum and at the bedside. Hence, the *expectant* or no treatment at all, was in the mouths of nearly every one, with earnest implorations to give but little medicine, "Oh! don't kill your patients with drugs, but let them alone." Time, treatment of time, is the best remedy. Thus contenting ourselves that our patients recover, and should death be the result, consoling ourselves that remedies could not arrest the disease nor prevent the fatal complications.

Permit me again to notice the words of Wood—"*Con-*

*trolling influence of treatment and materially shorten.*" Now, if it is probable that the morbid impression can be very much modified, might it not be equally as probable that it can be more so, and so on, until we arrive at abortion? I certainly think so.

To enter into an elaborate discussion upon the nature of Typhoid Fever, would be stale and uninteresting, as it would not enlighten, or prove that my opinion in relation to the abortion of the disease is correct. It suffices however to say, that I believe it is primarily and for some days purely a nervous affection; and that the so-called characteristic lesion is the effect, and not the cause; in which opinion I am supported by BARTLETT and others. I must contend for the correctness of these opinions, for upon them is founded my treatment. Are they not correct? They are. For certainly the symptoms of the so-styled characteristic lesion does not exist at the onset of the disease, nor for several days; and often, even then, so slight that some are wont to say it (the lesion) is not essential to the disease.

I do not believe that an affection of the intestines is necessary to constitute the disease. That is, I mean to say, that inflammation of the mucous follicles or disease of Pyer's glands will not exist because it is Typhoid Fever, but rather because the disease has not been arrested. For, if the perverted innervation be corrected in the first 48 or 60 hours, or perhaps after a longer period, while it is but a kindling spark, the affection of the bowels will not follow, but vice versa.

I have certainly succeeded in arresting this so much dreaded malady before any traceable, or even suspicious, symptoms of intestinal disease supervened. Some will no doubt argue, from preconceived notions, that they were not cases of Typhoid Fever, because they did not run the usual course, but yielded to remedies. But I must be allowed to argue that they were, because they had the same symptoms in the onset that others had that did run the usual course, and that in the same family, and under precisely the same circumstances.

Some of my professional friends suggest that this doctrine of abortion conflicts with the theory of contagion, which, however, I am inclined to think is not the fact. But admit it; does it therefore follow that it is incorrect? If so, facts do not establish theories, but theories facts, which is opposed to the spirit of the age, and orthodox medicine.

I believe, as a general rule, that nearly all contagious or infectious diseases are more or less of a specific nature, and also run, more or less, a specific course. But cannot many of them be very much shortened, or even aborted? They certainly can.

Syphilis, when in its earliest stages, can be aborted, which is a familiar fact to every tyro in the profession. It can also often be checked when somewhat advanced, and held at bay, rendering its effects harmless. It is the type of contagious diseases, and if let alone, and permitted to go on in its course uninterrupted, it pervades the whole system, committing ravages upon the whole human fabric.

Typhoid Fever is a contagious or infectious, and, primarily, only a nervous disease, and which, if permitted to take its course, pervades the whole system, committing ravages upon the whole human fabric. Now, if Syphilis be contagious, and will run its course if untouched, yet can be checked or aborted, why should not Typhoid Fever, a contagious disease, be also checked or aborted?

All contagious or infectious diseases are so by effluvia or poisonous emanations arising from the infected individual, or other source of the poison, coming in contact with the living tissue, either by inhalation or otherwise, the effect of which is to produce symptoms analogous to the disease from which it emanated; which symptoms are, for the most part, nervous, at least it is the case with Typhoid Fever. All contagious or infectious diseases have a stage of incubation and initiatory fever. Hence, I can perceive that the opium may act as a barrier to the further progress of the poison, either through some unknown chemical influence, or by its dynamical properties rendering the nervous system incapable of further morbid impressions, or by inducing that state in which it is enabled to throw off the effects of the poison.

The treatment by which I propose to abort this disease is, with full doses of opium. But when I say full doses, I do not mean the full dose as laid down in the books, but a four, six, or eight grain dose, or as much more as the severity of the case seems to require; that is, if that quantity is not sufficient to quiet thoroughly, I increase it every six or eight hours until I accomplish the desired end—to wit, to procure complete rest, sweating, etc. Less than four or six grains is worse than useless, as the symptoms of nervous origin will generally be aggravated instead of being mitigated. I have never, as yet, seen any bad results arising from the use of the large dose of Opium in Fevers of any kind, even when the cerebral symptoms were high and preponderate. The very symptoms that old and long established or acknowledged principles would teach, and have taught, contraindicated the use of Opium at all, are the very symptoms that seem to be the best managed by the larger or largest dose.

The same treatment I find well adapted to the disease if advanced to that stage in which it cannot be cut short. It will quench the burning flame even after it has scorched the boards, and make way for the more speedy repair and healing of the inflamed and ulcerated glands. I give it so soon as I see my patient, if the urgency of the case requires immediate medication. But I much prefer giving it at night, as the night is more favorable to the operation of all alterative medicines. Opium is, in this case, prescribed as an alterative to the nervous system. And another reason why I prefer to give it at night is, that I always order the hot pediluvium, and associate with it some hot drink—the tea of Eupatorium Perforiatum is perhaps the best—as it favors immediate action upon the cutaneous surface. Any hot drink might answer as well by adding a small quantity of Ipecacuanha to the Opium. I also enjoin quietude and darkness, in a good bed, and protection from draughts of cold air; all of which requisitions can be much better complied with at night than in the day, and the results are more satisfactory. If a patient is permitted to get up and be exposed to a cool draught of air while under the influence of a full dose of Opium, diaphoresis ceases, headache, giddiness, and sick stomach supervene, which, however, is all avoided by remaining covered up in bed. I repeat the dose at intervals of four, six, eight, or ten hours, as the particular case may seem to require, and continue the same as long as desired, say from two to four days, or even longer. The symptoms of each case must be the guide upon that point. I have frequently permitted the bowels to remain unmoved for five days, after which time I think it proper, in some cases sooner, that the bowels should be moved, to rid them of accumulations of fecal matter, etc., and that by a gentle enema. The same course should be pursued if, after the bowels have been evacuated, the disease does not seem to have given way thoroughly. It will sometimes be found necessary to increase the dose to sustain the necessary impression. I am speaking now in relation to the treatment of adults; nevertheless I do not hesitate to use the proportionate dose in children as young as four or five years of age. If the disease should run, more or less, its usual course, I use the same great remedy, adding, however, some of the usual remedies, as the neutral mixture of terb, etc., and I find the latter much more effectual with than without it —the "*Magnum donum Dei ad Homines.*"

The philosophy or precise *modus operandi* of this bold treatment I know not. It must be left for future investigation to determine. I do know, however, that it gives a complete quietus to the raging fever, soothes and hushes inquietude, lullabies the fretful ganglions and cerebral lobes into a pleasant and sweet repose, I cannot say slumber, but a short and refreshing sleep, often to awake in thoughts and feelings most sublime. I furthermore know that profuse diaphoresis nearly always follows the use of ths large dose of Opium, and more especially when something is combined to excite an immediate action upon the skin.

My worthy and talented friend, O. P. Hill, M. D., a gentleman of rare genius and accomplishments, formerly my partner in the practice of medicine, has tried the abortive plan, and believes that it will very materially shorten, if not abort entirely. Several gentlemen in this vicinity have tried it, and have expressed themselves as being well pleased with its effects.

I will here take occasion to speak of the valuable article of Dr. Anson G. Henry, of Springfield, Illinois, (see *Western Journal of Medicine and Surgery*, for January, 1852,) as it contains important truths, which, if more generally practised, would save many an ounce of precious blood, and many a dose of mercurial physic—both of which are so dangerous and detrimental to the predisposed consumptive. Phthisis, the great destroyer of human life, and the dread of all mankind, is ever ready to seize upon its victim; and a more unfavorable opportunity never offers than when the blood is rendered anemic by heavy draughts, or impoverished and poisoned by mercurial salivation.

I do not wish to be understood as arraying myself in opposition to these great and potent remedies, but simply to call attention to the excessive use of them, and also to the fact that they might, to a very great extent, be dispensed with by the adoption of Henry's Opium practice.

Should this treatment answer in the hands of others, as it seems to have acted in mine, it will prove to be a most valuable addition to the great art of healing, for which the profession will be greatly indebted to the above article upon Opium, by Dr. Henry.

My attention was first called to the use of the large doses of Opium in fevers by Prof. Rogers, while attending a course of lectures at the University of Louisville, during the winter of 1849–50, and also in 1850–1, who, in his notice of it, I think, styled it the novel practice of Dr. Henry.

Dr. O. P. Hill commenced the use of the large dose of Opium, in Dysentery, during the summer of 1851, rather by chance; being pressed with business, he gave it to some notorious patients to gain time; they immediately recovered, and which he profited and practised likewise, as he says, without respect of persons. I have been in the habit of using Opium freely, but cautiously, ever since I commenced practice, and have, since the publication of Dr. Henry's article, been using his four and six grain doses in all fevers, and most inflammations, and I have no cause to abandon the practice; but, upon the contrary, my most sanguine expectations have been more than realized. In Periodical Fever its use has invariably been crowned with success in my hands.

In Typhoid Fever I used it pretty freely, but did not presume that it could be aborted. But from the fact that my cases seemed to recover so much sooner than I was in the habit of seeing patients recover with the same disease under the usual treatment, I imagined that if it could be so perceivably alleviated and abridged that it might be entirely cut short; and I determined, to test it the very first good opportunity that offered. An opportunity soon offered, and I am much pleased with the result. One great advantage of this treatment, if only equally successful, is, that patients are infinitely less trouble to their nurses.

It might, perhaps, be well to give a short history of the cases that occurred in the family in which I tested the virtue of the treatment.

The residence of the family is healthy, and located in the country. Typhoid Fever is the common disease of the neighborhood, in fact any other form of fever is very rare. There were some eight or ten cases occurring successively, so that I was enabled to observe each case more closely. I will commence by noticing a case that occurred some two weeks before the other cases.

The case alluded to was a negro girl, about twenty years of age, and which only came under my observation to have a tooth extracted, which had been aching for two weeks or more. My attention was drawn to her appearance; she looked very much like one who had been quite ill, which I remarked, and her mother replied that she had, and that she believed the tooth only ached in consequence of her illness. She had been complaining all this time of head, jaw, tooth, and back ache; loss of appetite and general debility were the most prominent symptoms. Her bowels were not much inclined to diarrhea if let alone, but were very much so after the administration of the mildest aperient. After extracting the tooth, I gave Opium and Quinine in full doses; the object of which was to procure sleep, and equalize the circulation, as she had not slept well for some weeks, and her extremities were disposed to be cool. In fact, I treated the case for what I saw, and not for Typhoid Fever; for perhaps I never would have recollected it, except that there were other cases followed it.

The first case following was a negro boy, about twenty-two years of age. He was taken on the 21st of January, 1853, with very slight chilliness, loss of appetite, pain in the head, back, and limbs, and a general indisposition, and feeling of discomfort, though he would still go about. These symptoms continued for several days, and were followed by those indicative of disease of the alimentary canal, and also those especially characteristic of the disease of which we are treating, namely, tenderness of the right illiac region, tongue red at its tip and edges, with a whitish fur in the middle.

About the eighth or ninth day, at which time I saw him, the abdomen became quite tympanitic, pulse 140 per minute, rather full and irritable, skin hot and dry, sordes upon the teeth, drowsiness, sleeplessness, tongue cleaning and recoating, etc. Under the use of the large dose the pulse came down regularly ten or fifteen every twenty-four hours, diaphoresis was fully established, the tympanites very materially subsided, and sleep, with complete rest, was thereby procured.

On the 14th or 15th day convalescence is fully established, and attending, which the glands of the neck inflamed, and a troublesome abscess formed, which was resolved, and he convalesced steadily but slowly.

The second case was also a negro boy. He was taken about the same time, and the general symptoms were nearly the same as the first, except that it was more of the asthenic type. General soreness and debility of the entire muscular system were prominent symptoms. Tympanites was also a very prominent symptom, with exquisite tenderness. The discharges from the bowels were not very frequent but liquid, and of a new-cider appearance, which was also the case with the first. In this case an extensive and troublesome bed sore formed upon the sacrum. The treatment was nearly the same, and was equally efficacious.

Third, fourth, fifth, sixth, and seventh, of different ages, were taken while I was treating the two first, and were taken as near as could be with the same prodomic and initiatory symptoms. I instituted the abortive treatment, and they recovered in four or five days.

The eighth case following was a negro child, four or five years old, and was taken on the 22d of February, after all the others were dismissed. The family, as they say, regarded it as cold and worms, and consequently I did not see it for some days—March 2d—at which time, I did not attempt to abort it. So soon as I saw it, I was struck with that dull, melancholic, inexpressible, mystified and gloomy expression of countenance, which was, however, marked in all the other cases. The symptoms were very nearly the same as in the two other cases that run through all the various and diversified symptoms of the disease, and with the addition of hemorrhage of the bowels. In this case I treated the symptoms so that it might develop itself fully. Every person that saw her was satisfied that she had Typhoid Fever. Now the question is, if the two first and the last were Typhoid Fever, is it not presumable that the intervening cases were also?

I have written this article at the solicitation of divers of my professional brethren, who were familiar with my practice with opium; and if I am in error, I am so with all sincerity of purpose, and am open for conviction.

August 22d, 1853.

